# In silico characterization of multiple genes encoding the GP63 virulence protein from *Leishmania braziliensis*: identification of sources of variation and putative roles in immune evasion

**DOI:** 10.1186/s12864-019-5465-z

**Published:** 2019-02-07

**Authors:** Artur L. Castro Neto, Adriana N. A. L. M. Brito, Antonio M. Rezende, Franklin B. Magalhães, Osvaldo P. de Melo Neto

**Affiliations:** 1 Universidade Federal de Pernambuco, Recife, Pernambuco Brazil; 2 Instituto Aggeu Magalhães, Fundação Oswaldo Cruz (Fiocruz-Pernambuco), Recife, Pernambuco Brazil; 3 Centro Universitário Tabosa de Almeida – ASCES/UNITA, Caruaru, Pernambuco Brazil

**Keywords:** Leishmania braziliensis, GP63, Virulence proteins

## Abstract

**Background:**

The leishmaniasis are parasitic diseases caused by protozoans of the genus *Leishmania*, highly divergent eukaryotes, characterized by unique biological features. To survive in both the mammalian hosts and insect vectors, these pathogens make use of a number of mechanisms, many of which are associated with parasite specific proteases. The metalloprotease GP63, the major *Leishmania* surface antigen, has been found to have multiple functions required for the parasite’s survival. GP63 is encoded by multiple genes and their copy numbers vary considerably between different species and are increased in those from the subgenus *Viannia*, including *L. braziliensis*.

**Results:**

By comparing multiple sequences from *Leishmania* and related organisms this study sought to characterize paralogs in silico, evaluating their differences and similarities and the implications for the GP63 function. The *Leishmania* GP63 genes are encoded on chromosomes 10, 28 and 31, with the genes from the latter two chromosomes more related to genes found in insect or plant parasites. Those from chromosome 10 have experienced independent expansions in numbers in *Leishmania*, especially in *L. braziliensis*. These could be clustered in three groups associated with different mRNA 3′ untranslated regions as well as distinct C-terminal ends for the encoded proteins, with presumably distinct expression patterns and subcellular localizations. Sequence variations between the chromosome 10 genes were linked to intragenic recombination events, mapped to the external surface of the proteins and predicted to be immunogenic, implying a role against the host immune response.

**Conclusions:**

Our results suggest a greater role for the sequence variation found among the chromosome 10 GP63 genes, possibly related to the pathogenesis of *L. braziliensis* and closely related species within the mammalian host. They also indicate different functions associated to genes mapped to different chromosomes. For the chromosome 10 genes, variable subcellular localizations were found to be most likely associated with multiple functions and target substrates for this versatile protease.

**Electronic supplementary material:**

The online version of this article (10.1186/s12864-019-5465-z) contains supplementary material, which is available to authorized users.

## Background

The leishmaniasis are parasitic infectious diseases caused by flagellated protozoa belonging to the genus *Leishmania*, family Trypanosomatidae, and which are transmitted by sandflies of the genera *Phlebotomus* or *Lutzomyia*. These diseases are found as two major clinical forms, named as cutaneous leishmaniasis (CL) and visceral leishmaniasis (VL), with a global incidence for each in the range of hundreds of thousands of cases per year [1]. Multiple *Leishmania* species are associated with the leishmaniasis and distinct species, closely related or not, are responsible for the disease in different parts of the world. Those belonging to the subgenus *Viannia* are restricted to the New World (including *L. braziliensis* and *L. guyanensis*), have evolved separately from better known species belonging to the subgenus *Leishmania* (*L. major*, *L. infantum*, *L. mexicana* and others) and are associated with the mucocutaneous leishmaniasis (MCL), a more aggressive variation of CL [2].

As successful pathogens, the various *Leishmania* species have developed effective mechanisms to escape the mammalian host immune response and proliferate [3, 4]. Some of these evasion mechanisms are dependent on proteases, which help ensure that the parasites can invade the mammalian tissue, survive, differentiate and multiply [5]. The GP63 protease, also known as leishmanolysin or major surface protease (MSP), was first discovered in 1980 as the major surface antigen of the promastigote form of many species of *Leishmania* [6]*.* It was later found to be bound to the cell membrane through a GlycosylPhosphatidylInositol (GPI) anchor and was also identified as an important virulence factor. This is a zinc-dependent metalloproteinase, which belongs to the peptidase family M8 and the metzincin class and includes conserved features such as the motif HEXXHXXGXXH and a pro-peptide located in the protein’s N-terminal region that renders the proenzyme inactive during translation and is removed during its maturation and activation. The GP63 proteins also include an N-terminal signal sequence which directs them to the endoplasmic reticulum and to the *Leishmania* secretory pathway [7, 8].

GP63 has been found to play multiple roles during *Leishmania* infection in mammals, starting in the extracellular environment where it acts inactivating the complement cascade, by cleaving C3b into iC3b. This inactivation prevents the formation of the membrane attack complex (MAC), despite allowing the opsonisation of the *Leishmania*, mediated by iC3b, and facilitating its phagocytosis. GP63 can also facilitate the binding of the parasite to the macrophage through fibronectin receptors, cleaving proteins from the host’s extracellular matrix. Within the macrophages, it also acts to reduce the production of TNF, IL-12 and nitric oxide, which contributes to the protection and survival of the parasite, and provides the *Leishmania* with a faster entry into the macrophage, through the activation of a host tyrosine phosphatase [7, 9, 10]. GP63 has also been shown to be released through exosomes into the extracellular medium and this may facilitate its uptake by the macrophage even before the internalization of the *Leishmania* parasite [11]. Lack of GP63 drastically reduces the *Leishmania*’s ability to establish and maintain an infection, since the hosts are more likely to induce an innate immunity inflammatory response [12]. Within the host cell cytoplasm, GP63 has been shown to cleave the transcriptional factor AP-1, which regulates the production of pro-inflammatory cytokines and nitric oxide by the macrophage [11, 13]. GP63 was also shown to be associated with the inactivation of the mTOR kinase, leading to the inhibition of protein synthesis in the macrophage and providing an ideal environment for the proliferation of the pathogen [14].

Early studies have shown that GP63 is more abundantly expressed in the promastigote stage of the *Leishmania* life cycle, the proliferative stage within the insect vector. This expression may peak during metacyclogenesis, when the parasite prepares to infect the mammalian hosts, and is subsequently reduced again upon differentiation into amastigotes, the intracellular stage that multiplies within the mammalian macrophages [7, 15, 16]. The abundant GP63 expression in promastigotes indicates relevant functions also in the insect vector, presumably needed for survival and proliferation. Indeed, a potential involvement in the degradation of protein components that would lead to the adhesion of the parasite in the insect gut epithelium has been shown [17, 18]. Due to its wide substrate specificity, GP63 may also perform a nutritional role for the parasite, acting as an endopeptidase [19, 20], or even protect the *Leishmania* against the insect defences [19].

Concerning the GP63 gene organization, there is a noticeable variation in the number of gene copies encoding these proteins among different *Leishmania* species. In *L, major* these genes are present in more than one chromosome and multiple copies have been detected arranged in tandem [21], with the same multi-copy arrangement also found in *L. infantum* and *L. braziliensis* [22]. Noteworthy, however, is the substantial increase in the number of gp63 genes reported for *L. braziliensis* and other species belonging to the subgenus *Viannia*, when compared with the subgenus *Leishmania*. This was reported in early studies [23–25] and has been confirmed more recently by results derived from a screening for cosmids harboring multiple GP63 genes from *L. braziliensis* [26], as well as by genome sequencing data for different *Leishmania* species [27–29]. No clear biological reasons are known, however, to explain this expansion in the GP63 gene copy number. Here, aiming to contribute further to the understanding of the role of GP63 in *Leishmania* pathogenesis in general but with a focus on *Viannia* species, we sought to investigate the GP63 gene expansion further, using a range of in silico tools. We started by better defining the extent of GP63 gene diversity in *L. braziliensis*, followed by an in-depth analysis of the similarities and differences between different genes from this and related *Leishmania* species. The GP63 genes were first grouped according to their chromosomal localization followed by phylogenetic comparisons between different trypanosomatid species. Further grouping according to sequence similarities or differences within non-coding and coding elements was also carried out, in order to define putative functional distinctions. Possible mechanisms associated with the gene expansion due to DNA recombination were then investigated and variations in sequence mapped on the GP63 structure and linked with predictions of immunogenic potential. Our results are consistent with a selective expansion of a subset of GP63 genes in *L. braziliensis* that might be linked to mammalian pathogenesis and might be required for a better protection against the host immune system.

## Results

### Search for new *L. braziliensis* GP63 paralogs

The early studies based on hybridization assays [23, 24] had suggested that the total number of GP63 genes found within *Leishmania* species belonging to the *Viannia* subgenus is greater than the number of genes available at the TriTrypDB database and identified after the *L. braziliensis* genome sequencing and annotation. Recent data based on next generation sequencing have also suggested major variations in copy number of GP63 genes between species within the same subgenus, *Leishmania* or *Viannia*, that have not yet been included on the annotated genomes [28–30]. Here, to begin to understand the true diversity of the *L. braziliensis* GP63 genes, we first sought to reevaluate the available *L. braziliensis* GP63 gene sequences considering that the automatic annotation methods might have missed further genes. We therefore performed a reanalysis of the *L. braziliensis* genome sequences and searched for possible new GP63 paralogs that might not have been annotated. To do this we performed a search in the *L. braziliensis* genome using the Hidden Markov Models (HMMs) methodology [31], carried out after a grouping of the entire proteome set from different *Leishmania* species (described in methods). Nine subsets of GP63 sequences were created using the OrthoMCL tool in order to group these sequences and allow the search to be performed, as shown in Additional file 1: Table S1, with the number of genes in each subset varying in size from 56 to only two. All nine subsets were used to build HMMs and these were then applied for the search of new paralogs in the predicted proteome from *L. braziliensis* 2904. In general, all HMMs were able to find the GP63 sequences assigned to each subset, however no new paralogs were found during the search. The genome of *L. braziliensis* strain 2904 deposited on TriTrypDB lists 39 GP63 genes and, in total, the HMMs identified the presence of 38 related sequences. A single gene (LbrM.10.1720) was not recovered using these models and indeed its coding sequence did not provide an alignment with a score high enough to be considered as a GP63. The results of the search for each HMM are summarized in Additional file 2: Table S2 and confirm the gene count number for GP63 genes derived from the *L. braziliensis* genome sequencing, 38 genes, lower than earlier estimates based on the hybridization studies [23].

**Fig. 1:**
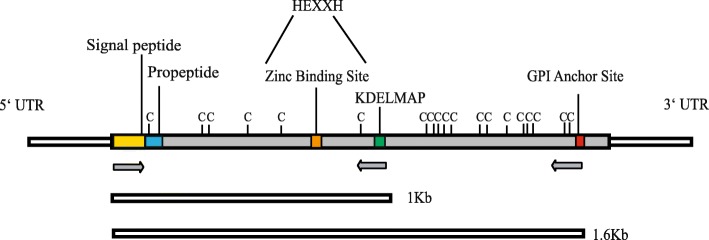
GP63 general gene features. The scheme highlights conserved elements found on most GP63 sequences, such as the signal peptide, propeptide, zinc-binding motif [8], multiple cysteine residues, the GPI anchor site and a nearly universally conserved motif of seven consecutive amino acids we named KDELMAP. The PCR strategy used to amplify the GP63 genes is also shown with products varying from 1 Kb (up to the KDELMAP region) and 1,6 Kb (finishing on the GPI anchor site)

Next, we considered that the shot gun nature of the sequencing strategy used for the assembly of the best genome available from a *Viannia* species, from the *L. braziliensis* 2904, might have led to the grouping of similar GP63 genes together, causing in turn a reduction in the number of genes found. In order to obtain as many natural GP63 sequences as possible, and therefore have a clearer idea of the true number of genes present in the *L. braziliensis* genome, we opted to amplify these genes using primers directed to conserved regions of representative genes identified in the genome analysis. The PCR strategy used to amplify the GP63 genes can be seen in Fig. 1, superimposed on a schematic representation of a typical GP63. The scheme highlights conserved elements found on all GP63 sequences, such as the zinc-binding motif, multiple cysteine residues, the GPI anchor site and a nearly universally conserved motif of seven consecutive amino acids we named KDELMAP. Six oligonucleotides annealing to sequences encoding the N-terminal ends of the GP63 sequences were used as 5′ primers, considering the variation previously observed within the N-terminus of the various GP63 genes and in order to maximize the number of genes amplified. As 3′ primers, two sets of two oligonucleotides annealing to the more conserved KDELMAP or the GPI anchor site motifs were alternatively used. Individual PCR reactions were set up with different pairs of oligonucleotides, always with a single 5′ and a single 3′ primer. After amplification, cloning and sequencing a total of 40 different GP63 gene fragments were obtained, with thirty-four of those having sequences different from the ones described in the databases. The new GP63 DNA fragments obtained by PCR are listed in Additional file 3: Table S3, which also includes the set of oligonucleotide primers used to amplify each sequence. In all 31 new gene fragments were found, since some were duplicates (also indicated in the table). The new sequences have all been submitted to GenBank and were compared with the already known GP63 genes from *L. braziliensis* and these analyses will be discussed further below. They are consistent with a higher copy number for the *L. braziliensis* GP63 genes than predicted based on the genome sequencing alone and more in agreement with the original estimates based on Southern-blots, although no precise quantification is possible either way. Despite the fact that both the genomic and PCR data used for our analyses are derived from the same *L. braziliensis* 2904 strain, the question of culturing in different laboratories being responsible for the differences observed regarding gene copy number and identification between the two sets of results can be raised. Nevertheless, considering the limited time frame for the culturing procedures, these could only have any impact on gene or chromosomal duplication events and would not lead to the different gene sequences that were found through PCR and/or DNA sequencing.

### Genomic analysis of known *Leishmania* GP63 genes

**Fig. 2:**
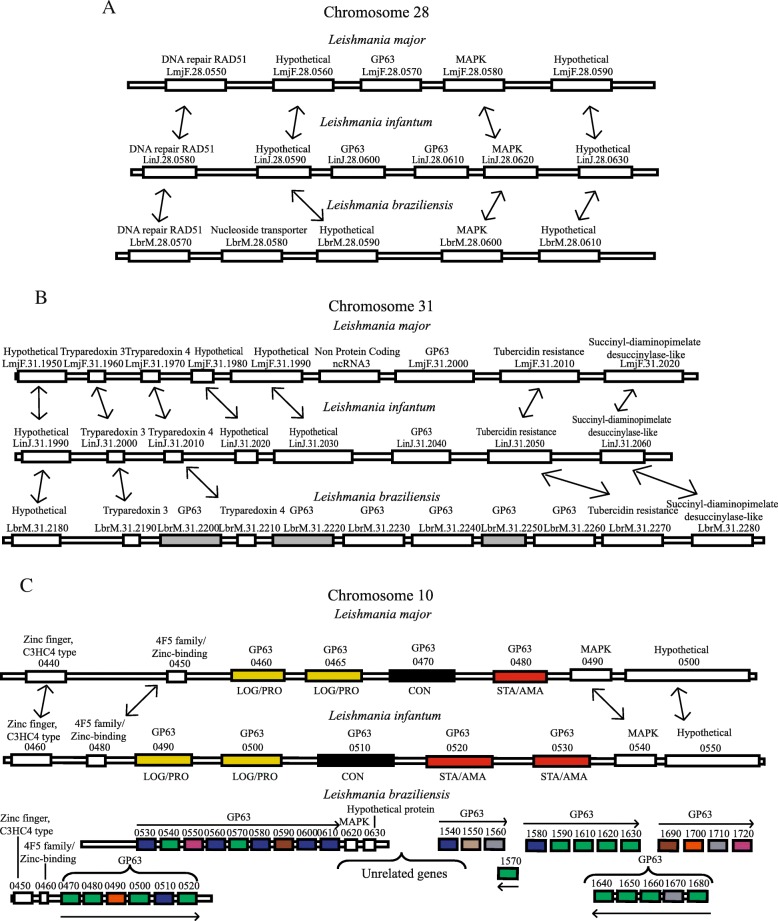
Genomic organization of *Leishmania* GP63 genes. Genomic organization of GP63 genes from *Leishmanis sp.*, showing the general distribution, location and synteny of these genes along chromosomes 10, 28 and 31. **a** Synteny analysis on chromosome 28 showing the localization of the GP63 gene in *L. major* and *L. infantum* and its absence in the equivalent position from the *L. braziliensis* chromosome 28. **b** Distribution of GP63 genes in *Leishmania sp*., chromosome 31. Pseudogenes from the *L. braziliensis* genome are highlighted in gray. **c** Expansion of GP63 genes in the *L. braziliensis* chromosome 10, when compared to *L. major* and *L. infantum*, within the same chromosome position, as indicated by the flanking genes on the left (some unrelated genes are also included). The horizontal arrows indicate the transcription sense of the genes and the yellow, black and red colors define the 3’ UTR groups identified for the *L. major* and *L. infantum* genes. The diagram for *L. infantum* does not include the preliminary annotation derived from the recent resequencing of its genome [30]. For the *L. braziliensis* genes, identical colors group those with similar 3’UTRs, as classified in Table 1

To clarify the relationship between the multiple GP63 genes in *Leishmania*, we opted to review their chromosomal organization within the major lineages of pathogenic *Leishmania*. For *L. major*, the best studied of the available *Leishmania* genome sequences, three sets of GP63 genes were found distributed in chromosomes 28 (one gene), 31 (one gene) and 10 (four genes) [21]. Based on the sequences available at the Tritryp database, a similar organization is also observed for *L. infantum* (summarized in Fig. 2) and *L. mexicana* (not shown), represented by two and one genes for chromosome 28 in *L. infantum* and *L. mexicana*, respectively, and one gene for chromosomes 31 in both species, considering that the *L. mexicana* chromosome 30 is equivalent to the *L. major* chromosome 31. Five GP63 genes are also found in chromosome 10 for both species and in agreement with previously reported data for *L. infantum* [22].

In *L. braziliensis*, based on the available genomic data for the 2904 strain, major differences in the organization of the GP63 genes are observed when they are compared with those found in species from the *Leishmania* subgenus. First, no GP63 gene is found on chromosome 28, as highlighted before for other *Viannia* species [32], despite the presence of orthologues to the same genes flanking the single GP63 sequence from *L. major* and *L. infantum*. In contrast, six GP63 genes or gene fragments are found on chromosome 31, again generally flanked by orthologues to the same genes found flanking the GP63 gene found in the *L. major* and *L. infantum* chromosome 31. Even more noteworthy, however, are the 33 GP63 genes found clustered on chromosome 10. Again, these are localized to the same region seen harboring the other *Leishmania* chromosome 10 genes, as confirmed by the presence of neighboring sequences encoding orthologues to those found flanking the *L. major* and *L. infantum* GP63 genes from chromosome 10. However, the precise gene organization cannot be properly defined and many of the genes sequenced are assembled in relatively short contigs, as indicated in the scheme from Fig. 2c. Again, this might be due to the high similarity between the gene sequences and the nature of the sequencing strategy which might have prevented a proper assembly of repeated sequences.

The significantly greater number of *L. braziliensis* GP63 genes from chromosome 10 is supported by our PCR data where primers sets directed to the chromosome 10 genes were able to amplify more genes than the ones originally used for their synthesis. For example, a primer pair designed to amplify the gene LbrM.10.0470 allowed the amplification of eight different gene fragments (G0510B2; G0560B1; G0560B2; G1610B3; G1610B4; G1610B5; G1610B6; G1620B1) and similarly, the primer pair directed to gene LbrM.10.0540 amplified fragments from six different genes (G0510C1; G0510C2; G0540C1; G0560C4; G1640C1; G1640C2). In contrast, two sets of PCR reactions directed to a single GP63 gene from chromosome 31, using the same 5′ primer and two distinct 3′ primers, only led to the amplification of the same gene, LbrM.31.2260 (Additional file 3: Table S3). Indeed, we believe that most of the six GP63 genes annotated from the *L. braziliensis* chromosome 31 might not exist and in fact are either pseudogenes or derived from genome assembly errors. Only one of those genes (LbrM.31.2260) has GP63-related protein features, such as the propeptide domain (HEXXH), and shares a high similarity (85%) with the *L. major* and *L. infantum* chromosome 31 genes. The LbrM.31.2200, LbrM.31.2220, LbrM.31.2230, LbrM.31.2240 and LbrM.31.2250 genes have stop codons in the middle of their sequences and/or in alignments showed identical N-terminal or C-terminal regions to LbrM.31.2260 (data not shown). It is possible that these genes may represent parts of LbrM.31.2260 not properly assembled and this in agreement with our PCR data finding only LbrM.31.2260. Overall these results are consistent with the expansion in the number of GP63 genes in *L. braziliensis*, and other species belonging to the *Viannia* subgenus, being mainly directed to the chromosome 10 genes.

### GP63 evolutionary analyses

**Fig. 3:**
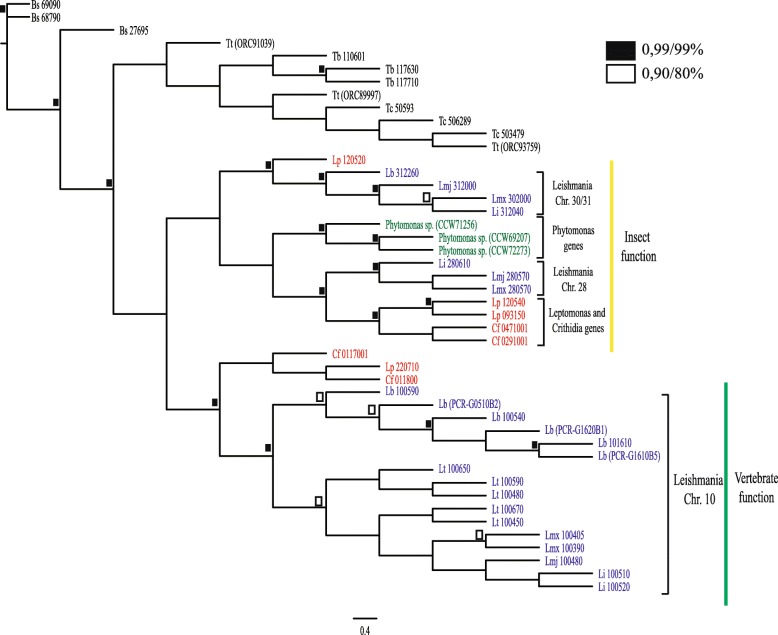
Phylogenetic tree comparing selected *Leishmania* GP63 paralogs with sequences from more distantly related organisms. The tree demonstrates the proximity between the GP63 genes from the *Leishmania* chromosomes 28 and 31, with GP63 genes from monoxenes trypanosomatids genes, such as *Leptomonas pyrrhocoris* and *Crithidia fasciculata*, as well as, genes from a plant tripanosomatid, like *Phytomonas sp.* Values for highly supported nodes have been replaced by black and white squares, which represents the Bayesian posterior probabilities and bootstrap support for PhyML, respectively. Bs: *Bodo saltans*, Tt: *Trypanosoma theileri*, Tb: *Trypanosoma brucei*, Tc: *Trypanosoma cruzi*, Lp: *Leptomonas pyrrhocoris*, Cf: *Crithidia fasciculata*, Lb: *Leishmania braziliensis* M2904, Lmj: *Leishmania major*, Lmx: *Leishmania mexicana*, Li: *Leishmania infantum*, Lt: *Leishmania tarentolae*

Sequences encoding GP63 related genes are also found in other trypanosomatids and more distantly related kinetoplastids and these include multiple genes from *T. brucei*, *T. cruzi*, and others. The number of genes in these parasites is quite variable. *T. cruzi* has over 150 GP63 genes annotated in the TriTrypDB database, but with many pseudogenes among them. *T. brucei* and *C. fasciculata* have a smaller amount with 10 and 18 genes respectively. This multiplicity of GP63 genes along the various kinetoplastids lineages reinforce the multiple roles this protein has, independent of the life cycle of the organism involved. Here, we next sought to assess how the *Leishmania* GP63 genes are related to those found in more distantly related kinetoplastids and whether some function can be inferred based on which genes are found in each organism. To do this we built a phylogenetic tree comparing the most divergent and representative sequences from the three major sets of *Leishmania* GP63 genes (from chromosomes 10, 28 and 31) with genes from different *Trypanosoma* species (*T. brucei*, *T. cruzi* and *T. theileri*) and more distantly related organisms. These included species that parasitize reptiles (*L. tarentolae*) and plants (*Phytomonas sp.*), have monoxenous life-cycles in insects (*Crithidia fasciculata* and *Leptomonas pyrrhocoris*) and are free living (*Bodo saltans*). As shown in Fig. 3, the phylogenetic analysis could separate the GP63 genes mapped to the *Leishmania* chromosome 10 from those genes mapped to chromosomes 28 and 31. We also could observe a clear separation between the *Leishmania* subgenus based on the genes located on chromosome 10. Noteworthy are the *T. cruzi* and *T. brucei* GP63 sequences more closely associated with the GP63 genes from *B. saltans* and *T. theileri*. Also, when we observe the clustering of the genes present in chromosomes 28 and 31 from *Leishmania*, they generally show more proximity to the genes from *L. pyrrhocoris, C. fasciculata* and *Phytomonas sp*. Nevertheless, one *L. pyrrhocoris* and two *C. fasciculata* genes are more closely related to those from the *L. braziliensis* chromosome 10*.*

Overall, the gene clusters shown in the tree highlight the higher similarity between the *Leishmania sp.* genes from chromosomes 28 and 31 with the GP63 genes found in organisms that live in insects only or parasitize plants. For instance, the 38 annotated GP63 genes from *Phytomonas* are more closely related to the *Leishmania* chromosome 28 GP63. It is then possible to hypothesize that these genes might me more involved in the insect stage of the parasite life cycle. Genes more closely related to the chromosome 10 GP63 genes can be found in the insect parasites *L. pyrrhocoris* and *C. fasciculate*, but in general these genes seem to have suffered a substantial expansion within *Leishmania* species.

### Evaluation of the sequence diversity of the *Leishmania* GP63 genes from chromosome 10

**Fig. 4:**
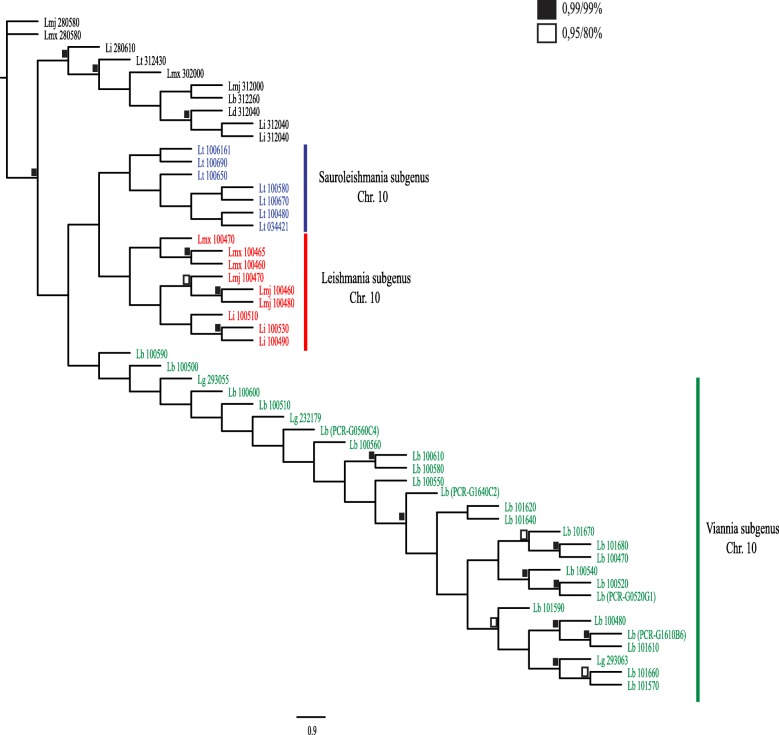
Phylogeny of *Leishmania* GP63 paralogs from chromosome 10. Phylogenetic tree comparing multiple chromosome 10 GP63 genes from selected *Leishmania* species. The tree highlights the separation of the GP63 genes according to the three main *Leishmania* subgenera (*Sauroleishmania*, *Leishmania* and *Viannia*). Values for highly supported nodes have been replaced by black and white squares, which represents the Bayesian posterior probabilities and bootstrap support for PhyML, respectively. Lb: *Leishmania braziliensis* M2904, Lmj: *Leishmania major*, Lmx: *Leishmania mexicana*, Li: *Leishmania infantum*, Lt: *Leishmania tarentolae*, Lg: *Leishmania guyanensis*

Considering the expansion of the chromosome 10 GP63 genes in the *Leishmania* lineage in general, and even more so in *L. braziliensis* and other *Viannia* species, we then opted to investigate the origins of their diversity further. To do this we compared the full extent of the chromosome 10 GP63 sequences from relevant *Leishmania* species using as outgroups selected genes from chromosomes 28 and 31. For this analysis, we also included sequences from *L. tarentolae* (based on the published genome sequence [33]), where a similar expansion in the chromosome 10 GP63 genes was noticed, with 49 genes found in this chromosome while only one gene was found in chromosome 31 and another in chromosome 33. *L. tarentolae* is currently classified within the *Sauroleishmania* subgenus, but it is likely to be more closely related to the *Leishmania* subgenus than to *Viannia* [2, 34]. The relevance in including the *L. tarentolae* sequences is due to the fact that it does not parasitize mammals, only lizards, meaning that any potential role in pathogenesis associated with the chromosome 10 GP63 genes is not dependent on their mammalian hosts. We also included in this analysis the new *L. braziliensis* sequences generated by us through the PCR approach. The phylogenetic tree shown in Fig. 4 summarizes the results from these analyses based on alignments using the full-length sequences for all proteins (or the full-length PCR fragments). For clarity, only the most divergent representative sequences were used to build this tree, with those very similar or nearly identical to the ones shown purportedly removed from the final figure. In the original analysis all chromosome 10 sequences from the selected species were used but with similar results (not shown). Within each of the three *Leishmania* subgenera analysed, all GP63 sequences from chromosome 10 are more closely related to sequences from the same or related species than to sequences found in species belonging to the other subgenera. Even within the *Leishmania* clades, the *L. infantum* genes (in red) seemed to be more closely related to each other than to their *L. major* counterparts, although for the two *Viannia* species (*L. braziliensis* and *L. guyanensis*) analysed genes (in green) more closely related between the two species were found. These results are in agreement with independent expansions on the number of the chromosome 10 GP63 sequences in each clade, with major expansions occurring for both *Sauroleishmania* (in blue) and *Viannia* species. For the latter species, at least, the start of this expansion may have preceded the split between *L. braziliensis* and *L. guyanensis* but has subsequently continued and may be an ongoing process.

### Identification of functional differences between the various *Leishmania* GP63 genes from chromosome 10

**Fig. 5:**
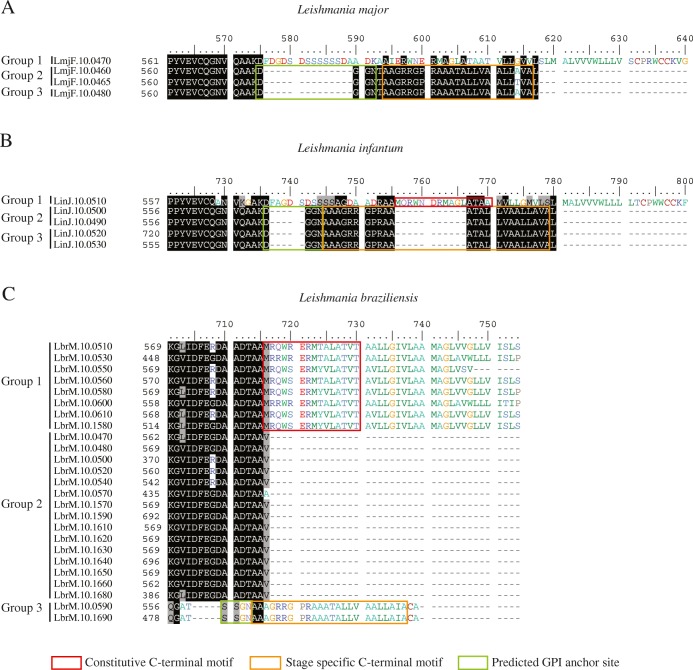
*Leishmania major*, *L. infantum* and *L. braziliensis* C-terminal alignments and groups. Alignment showing the difference in the C-terminuses of the chromosome 10 GP63 sequences having divergent 3’ UTRs. **a** and **b** shows the *L. major* and *L. infantum* C-terminus sequences, respectively, their groups based on their expression profiles and the predicted GPI anchor site (boxed in green). The motif identified in the constitutively expressed *L. infantum* group 1 sequence also found in the *L. braziliensis* group 1 C-terminus is boxed in red, while the motif found in the *L. infantum* and *L. major* stage specific genes from Groups 2 and 3 nearly identical to a similar motif from the *L. braziliensis* group 3 genes is boxed in orange. **c** Alignment of the C-terminal ends of the different *L. braziliensis* chromosome 10, with the grouping described in the text and the conserved elements also seen in *L. major* and *L. infantum* indicated as for A and B

So far not much is known regarding possible functional differences between the various GP63 genes found within any specific trypanosomatid. In *Leishmania*, with the goal of defining functional differences between multiple GP63, and even prior to the completion of the first *Leishmania* genomes, early studies investigated the expression pattern of selected genes attempting to identify differences in expression during the parasite life cell cycle [8, 15, 35]. In *L. infantum* and *L. major*, three growth stage-specific patterns of expression were observed for the then known GP63 genes, with one gene constitutively expressed, a second gene (or genes) expressed during the log phase of promastigote growth and the third gene expressed only in stationary phase cells and/or amastigotes [15, 25]. Here, by comparing their coding sequences and 3’ UTRs with those from the available genomes, we were able to map those genes within the annotated genome sequences (indicated in Fig. 2): LinJ.10.0510 and LmjF.10.0470 are the constitutively expressed genes (here named Group 1 – colored in black in the figure); LinJ. 10.0490/LinJ.10.0500 and LmjF. 10.0460/LmjF.10.0465 are equivalent to the previously described log phase promastigote genes (Group 2 – highlighted in yellow); LinJ. 10.0520/LinJ.10.0530 from *L. infantum* and the *L. major* LmjF.10.0480 gene correspond to the stationary phase/amastigote specific GP63 (Group 3 – colored in red). The Group 1 genes are characterized by unique 3’ UTRs and 3′ intergenic regions absent from the remaining chromosome 10 genes, while the Group 2 and 3 genes share very similar sequences within the first ~ 400 nucleotides of their 3’UTRs, although these subsequently diverge into two distinct patterns that correlate with the two groups (these genes and their groups are highlighted by different colors in the scheme from Fig. 2). We also looked at differences within the coding sequences that could be typical of GP63 genes belonging to any particular group. As previously reported for *L. major* [25], a clear distinction is observed between the C-terminus of the Group 1 proteins and those from Group 2 and 3. Both *L. major* and *L. infantum* Group 1 proteins are characterized by a longer C-terminus enriched in hydrophobic and positively charged residues and lack the typical asparagine required for the GPI anchor. In contrast, the shorter C-terminus from the Group 2 and 3 proteins include the GPI anchor site and end in a stretch of mostly hydrophobic amino acids (Fig. 5).

Next, we attempted to group the *L. braziliensis* chromosome 10 genes based first on similarities and differences within the putative 3’UTRs. Thirty genes were analyzed based on the sequences available from the reference genome sequence and these were classified into six groups according to their 3’UTR, with the first two groups represented by 8 and 15 genes, respectively, while the remaining groups included only one or two genes (Table 1 – also colored differently in Fig. 2). When these 3’UTRs were compared with the three *L. infantum* and *L. major* groups no clear similarities were found with any of the *L. braziliensis* groups, likely due to the large sequence variation observed between species from the two distinct subgenera. We also looked at the amino acid sequences, looking for features in common for *L. braziliensis* genes sharing similar UTRs. No association with features such as signal peptide, transmembrane domains and isoelectric points was found, however distinct C-terminus were observed which also separated the *L. braziliensis* into six groups, with a clear association observed between each 3’UTR group and nearly all of the proteins’ C-terminal ends (Table 1). Since Groups 4, 5 and 6 consists of truncated proteins they will not be considered further here, but for the remaining three groups their C-terminal ends were also compared with those seen to be associated with the *L. infantum* and *L. major* groups. A clear association between the two proteins from the *L. braziliensis* Group 3 and the *L. major*/*L. infantum* Groups 2 and 3 can be seen, since they share a nearly identical C-terminus that includes the GPI anchor attachment motif (DGGN [36]). The Group 1 genes from *L. braziliensis* also share conserved elements with the *L. major*/*L. infantum* Group 1, such as the lack of a typical GPI anchor site and the presence of a hydrophilic set of amino acids followed by a hydrophobic region resembling a transmembrane segment. The motif “MRQWRERMTALATVT” found in the *L. braziliensis* sequences is also very similar to the “MQRWNDRMAGLATAA” motif found in *L. infantum* LinJ.10.0510 gene. Only the *L. braziliensis* Group 2 genes then, characterized by a shorter C-terminus missing entirely the GPI anchor site or related hydrophobic sequences, do not seem to have counterparts in *L. infantum* nor in *L. major*. Nevertheless, it seems likely that, as observed in *L. infantum* and *L. major*, the different groups of *L. braziliensis* chromosome 10 GP63 genes are also differentially regulated during the parasite growth in culture and this is in agreement with the different 3’UTRs seen associated with each group.

**Table 1:** *Leishmania braziliensis* GP63 3’ UTR and C-terminal end gene groups. Table showing the Gp63 gene groups from chromosome 10 defined according to the 3’ UTR and C-terminuses sequence similarity

Group	*L. braziliensis* 3’UTRs	*L. braziliensis* chromosome 10 GP63 paralogs
1	LbrM.10.0510, LbrM.10.0530, LbrM.10.0560, LbrM.10.0580, LbrM.10.0600, LbrM.10.0610, LbrM.10.1540, LbrM.10.1580	LbrM.10.0510, LbrM.10.0530, LbrM.10.0550, LbrM.10.0560, LbrM.10.0600, LbrM.10.0610, LbrM.10.1540, LbrM.10.1580
2	LbrM.10.0470, LbrM.10.0480, LbrM.10.0500, LbrM.10.0520, LbrM.10.0540, LbrM.10.0570, LbrM.10.1570, LbrM.10.1590, LbrM.10.1610, LbrM.10.1620, LbrM.10.1630, LbrM.10.1640, LbrM.10.1650, LbrM.10.0600, LbrM.10.1680	LbrM.10.0470, LbrM.10.0500, LbrM.10.0520, LbrM.10.0540, LbrM.10.0570, LbrM.10.1570, LbrM.10.1590, LbrM.10.1610, LbrM.10.1620, LbrM.10.1630, LbrM.10.1640, LbrM.10.1650, LbrM.10.1660, LbrM.10.1680
3	LbrM.10.0590, LbrM.10.1690	LbrM.10.0590, LbrM.10.1690
4	LbrM.10.0550, LbrM.10.1720	LbrM.10.1720
5	LbrM.10.0490, LbrM.10.1700	LbrM.10.0490, LbrM.10.1700
6	LbrM.10.1550	LbrM.10.1550

### Gene recombination in GP63 sequences from chromosome 10

**Fig. 6:**
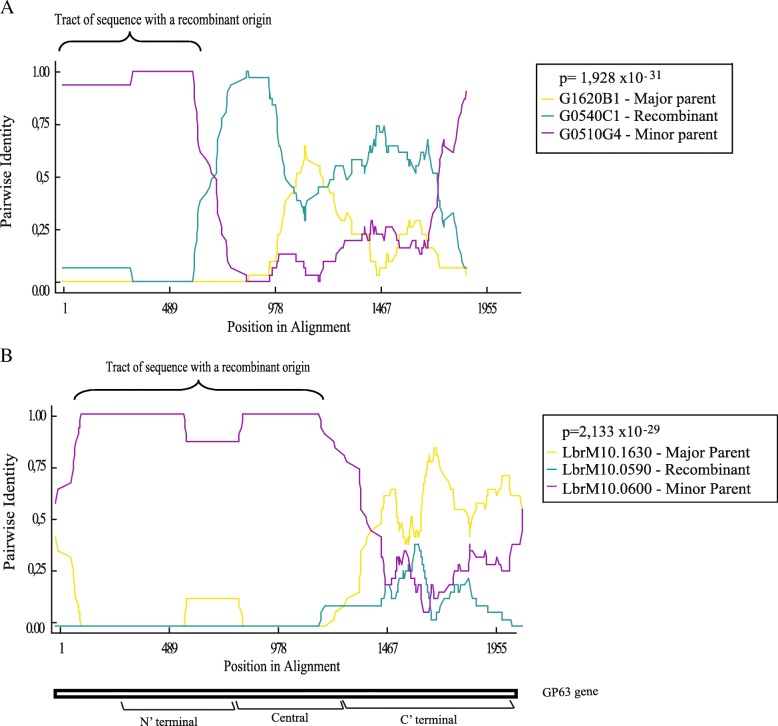
*Leishmania braziliensis* GP63 recombination events. Representative figure indicating recombination events identified using the RDP4 software. The *p*-values calculated for the recombination events are indicated as well as the parental and recombinant regions of the chosen gene. The graph shows the gene that underwent a recombination event (in blue) and other two genes that most likely were involved in this recombination, named major parent (in yellow) and minor parent (in purple), identified by pairwise alignment. Despite their definition as major or minor parent, any one of these genes may be involved in the recombination. **a** Predicted recombination event between two PCR genes, G1620B1 and G0510G4, with the recombinant gene (G0540C1) showing a 100% identity in most of the N-terminus region with G0510G4. **b** Predicted recombination event between two annotated genes, LbrM10.1630 and LbrM10.0600, showing the putative recombinant gene (LbrM10.0590) with an almost 100% pairwise identity in most of its N-terminal and central regions with the gene LbrM10.0600. A representative GP63 gene structure is shown in the bottom of the figure, discriminating N-terminal, Central and C-terminal regions

Through analyzes of the alignments generated in this study, we identified that specific regions of certain GP63 gene sequences were very similar to equivalent regions from other GP63 genes which otherwise were more divergent. For example, certain small motifs generally seen only on the *L. braziliensis* Group 1 genes were also found in one or more of the group 2 genes and vice-versa, an indication of gene recombination. Indeed, the locus for these genes is reported as having high plasticity [23, 37] and the data from the literature shows that this gene family can be influenced by mosaic or fragmental gene conversion [26, 38]. Here, in order to understand why the expansion of the GP63 genes occurs mainly on chromosome 10, we performed an *in-silico* search for recombination events targeting these genes so as to better evaluate whether their variability was related to intragenic recombination. The software chosen to find the recombination events (RDP4) uses several tools to determine events such as the likely position of recombination breakpoints and the identity of sequences most closely related to the gene being evaluated. In this study we only considered recombination events that were detected by at least two of the tools tested. Therefore, we decided to perform a gene recombination analysis with all the GP63 genes of *L. braziliensis* present in databases and the ones generated by us through PCR. We first targeted the chromosome 31 GP63 genes, but no recombination events were detected by the software. In contrast, when the 38 PCR sequences from chromosome 10 were analyzed 30 (or 79%) were reported as recombinant genes. Regarding the database genes, 32 recombination events were found, sometimes with more than one event for the same gene (Fig. 6 and Additional file 4: Table S4). Gene duplication and recombination events then are possibly the major source of the novel GP63 sequences seen in the chromosome 10 from *L. braziliensis* and closely related sequences.

### Protein structural modeling, mapping of variable regions and B-cell epitope prediction

**Fig. 7:**
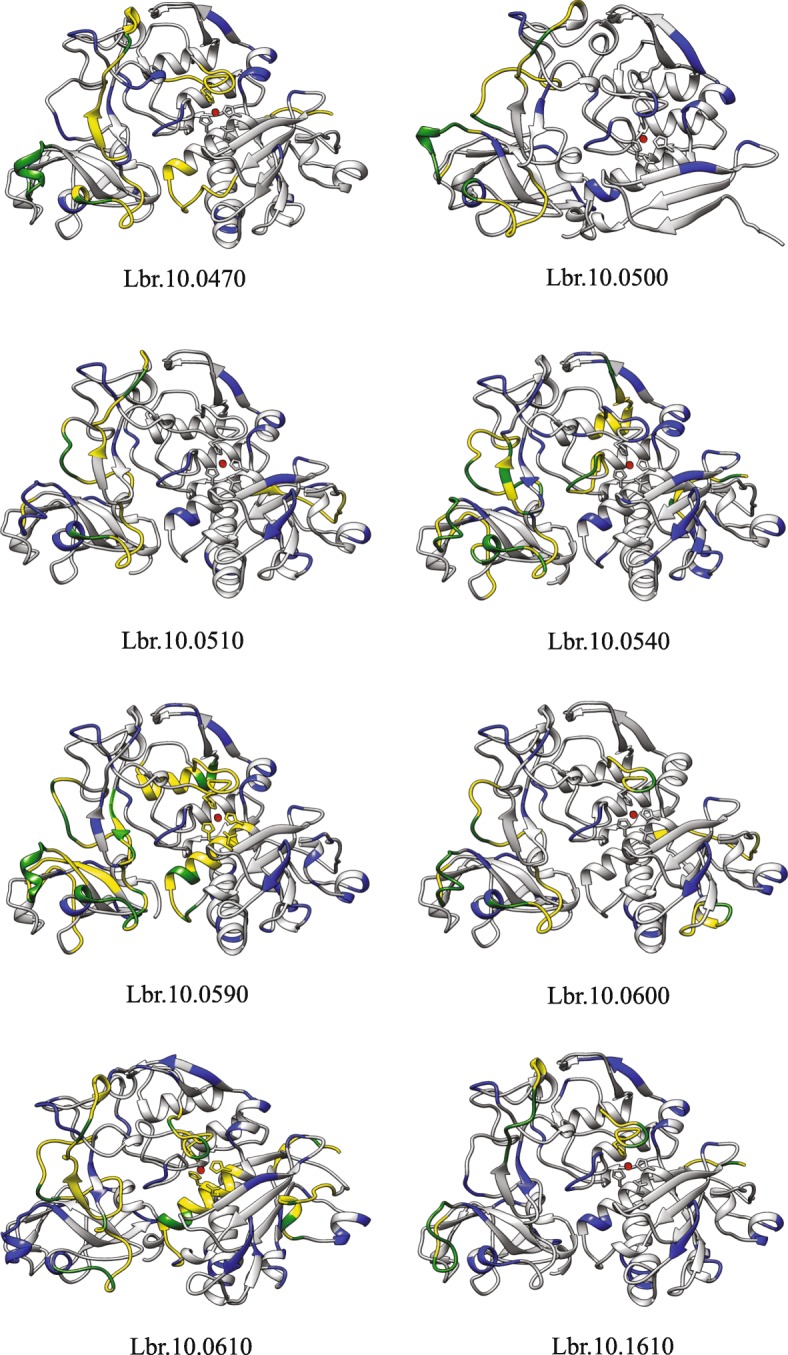
Comparative protein modelling of selected *Leishmania braziliensis* GP63 paralogs from chromosome 10. Modelled structures of eight representative GP63 paralogs from the *L. braziliensis* chromosome 10. The segments labelled in blue indicate the variable motifs in each protein, while the segments yellow represent the predicted linear B-cell epitopes and the green markings show variable motifs coinciding with the predicted epitopes

Based on the crystallized structure of a membrane GP63 from *L. major* promastigotes, GP63 was identified as a compact protein consisting predominantly of β sheet secondary structure elements divided into three distinct domains (N-terminus, Central domain and C-terminus) and with features typical of the catalytic modules of zinc proteases [39]. After observing the recombination events and sequence variations between the multitudes of *L. braziliensis* GP63 genes, we decided to investigate where these variations are found along the 3D structure of the protein. Through three-dimensional protein structure predictions, we were able to model the structure of eight divergent GP63 sequences with high modeling scores, as can be seen in Fig. 7. We then mapped on the models the most variable motifs identified by the previous multiple alignments (highlighted in blue in the structures shown in the figure). As can be observed, most of the variable regions were positioned externally on the structures.

We next sought to evaluate how the various GP63 sequence would be recognized by the B cells from the mammalian immune system and also to predict their ability to induce the production of specific antibodies. Linear and conformational B-cell epitope predictions were carried out using the various chromosome 10 GP63 sequences from *L. braziliensis*. The linear epitope predictions returned 56 epitopes from the sequences of the modeled proteins. Regarding their localization within the various structures, from the total of 56 epitopes, 41 were mapped to the proteins’ external regions, whereas six were localized internally and nine could not be evaluated due to the comparative nature of the tridimensional structure modeling (Table 2). The modeling is based on a mature GP63 crystallized structure, which lost part of its N-terminal region, during the protein posttranslational modification, which prevented the assessment of epitopes localized to this region. Out of the 41 linear epitopes localized externally, 33 coincide with motifs that display sequence variation, while 8 are found in regions conserved between the different GP63 sequences. Considering only the epitopes that were predicted to localize internally, four coincide with variable sequences while two were associated with conserved regions. As for the prediction of conformational B-cell epitopes, the analysis returned 40 epitopes, with 32 mapped to the proteins’ external region. Twenty-three of those were in motifs with sequence variation, while nine were in conserved regions. Regarding the eight epitopes localized internally, five coincided with variable sequence motifs and three were in conserved motifs. We also identified 14 motifs that were present in both linear and conformational epitope predictions (not shown). As above, most of the epitopes coincided with variable motifs localized externally, as shown in Table 2. Noteworthy also is the fact that a peptide from *L. infantum* GP63 that has been previously shown to react strongly with sera from dogs infected with visceral leishmaniasis [40] is nearly identical to one of the *L. braziliensis* B-cell epitopes predicted by our analysis within the Lbr10.0590 polypeptide (not shown). Overall, these results are consistent with variable regions localizing externally and being more capable of inducing an immune response.

**Table 2:** *Leishmania braziliensis* GP63 B-cell epitope prediction. Table showing the distribution of the predicted epitopes along the studied GP63 paralogs

Total Number of Epitopes	Conserved Region	Sequence Variation Region	Type of Epitopes	Protein Region
41	8	33	Linear	External
6	2	4	Linear	Internal
32	9	23	Conformational	External
8	3	5	Conformational	Internal

## Discussion

The in-silico analysis carried out here highlights the strong selective pressure for the expansion in copy number of the chromosome 10 GP63 genes within *Leishmania* species, and in particular in the *Viannia* and *Sauroleishmania* subgenera. The increased number of the chromosome 10 GP63 genes in different *Leishmania* species evolved independently generating a wide range of paralogs, which display sequence variations and may be generated by recombination. This expansion seems to be an ongoing process that might be related to pathogenesis or defense mechanisms directed to the vertebrate host and the parallel expansion in both *Viannia* and *Sauroleishmania* species is something that must be taken into account. Such expansion of multiple genes arranged *in tandem*, originating from duplication and recombination events, demonstrates the adaptability of *Leishmania* species to the environment, associated with the evolutionary pressure suffered by the GP63 genes [28]. As a result, the presence of these multi-copy arrays may lead to speciation [41] or indicate the possible need for stage-specific genes [28]. As previously highlighted [42], an expansion in GP63 sequences also occurred independently in other trypanosomatids, such as *T. cruzi* and *T. brucei*, and this led to novel GP63 domains which might be associated with species-specific or group-specific functions. It is likely then that this expansion in *Leishmania* GP63 genes might be related to novel aspects of the pathogenesis of these parasites to the vertebrate hosts, but this still needs to be better defined. When multiple strains from a single species is considered the overall GP63 diversity might be even greater, as recently evaluated [43]**,** and this might be associated with possibly different virulence phenotypes and clinical outcomes for the disease. The recent release of a new *L. infantum* genome based on data using two distinct methodologies of next generation sequencing, and showing a higher copy number of GP63 genes for this species [30], also highlights the need for better quality genomes in order to properly define the true diversity of these genes for multiple *Leishmania* and trypanosomatid species.

The expansion observed in the chromosome 10 genes are concentrated in the Group 1 and Group 2 genes and, if we extrapolate the expression for Group 1 based on the data with the *L. major* and *L. infantum* genes [15, 25], they are likely to be expressed constitutively with likely functions during the mammalian infection. In contrast, for the Group 3 genes, with only two paralogs, their expression might be restricted to the promastigote stage of the parasite life cycle, therefore with minor or no relevant function in the mammalian host. For all three groups their expression will have to be confirmed but the large sequence variations observed for both Groups 1 and 2 genes, concentrated on potentially immunogenic regions localized on the surface of the GP63 molecules, also imply related expression patterns during the mammalian stage of the *Leishmania* life cycle. As previously shown in *L. major* and *L. mexicana* [44], these expression patterns should be linked to the mRNA 3’UTRs and sorting out the molecular mechanisms associated will be a major endeavor. An important question that emerges regarding the expression of the genes from Groups 1 and 2 is related to the multiple paralogs. Are multiple genes belonging to the same group expressed simultaneously or some are expressed more efficiently than others or alternatively? This will also need to be investigated further.

Another relevant question remaining deals with the functional roles for the distinct groups and how these might be associated with sequence differences between the paralogs. A possible link with the proteins’ subcellular localization is presumed based on the differences in the C-terminus of the subsets identified and the presence or absence of a typical GPI anchor. These differences regarding the presence or absence of GPI anchor sites have been suggested before based on comparisons between the *L. major* and *L. infantum* GP63 sequences [8]. Here, the C-terminal Group 2 of *L. braziliensis* GP63 sequences lacks the GPI anchor signal entirely, which is consistent with proteins that are directly secreted into the extracellular medium, as previously reported for *L. mexicana* GP63 [45]. This release into the extracellular environment might contribute at the early stages of infection, due to the ability of GP63 to digest the extracellular matrix proteins, facilitating parasite mobility and invasion [46]. Alternatively, these proteins might be selectively transferred to exosomes and later to the macrophages in order to influence its metabolism and promote *Leishmania* growth [11]. For the *L. braziliensis* Group 1 proteins, they all share a C-terminus having a likely transmembrane domain with no clear GPI anchor site. Lack of a typical GPI anchor site however, with a more likely transmembrane domain identified, was also seen in the *L. major* Group 1 gene, which was nevertheless seen to have a GPI anchor [25]. The distinct C-terminal ends nevertheless clearly suggest critical differences in subcellular localization for the distinct GP63 groups, but these need further experimental confirmation.

In early studies performed with *L. guyanensis*, it was suggested that new GP63 genes may be generated by events of mosaicism through recombination between 5′ and 3’ UTRs and protein coding regions [24], and mosaicism in GP63 sequences was subsequently also found in *L. braziliensis* genes [26]. The data obtained by us corroborate with other studies investigating GP63 recombination that found it to target mainly the N-terminal and C-terminal regions of the gene [37, 38]. The impact of the GP63 sequence variability in its structure has been investigated in a wider scale, comparing *Trypanosoma* and *Leishmania* sequences, and found to be associated with variability in its zinc binding site and presumably activity [42]. In a recent study targeting *L. braziliensis* GP63 sequences, structural differences have also been found to be associated with sequence variability, implying functional differences, such as during substrate binding, which may affect the interaction with the host [47]. Alternatively, the variability in protein structure could mainly affect recognition by the host immune system and promote infection mainly because the host would need to produce different antibodies to neutralize a single group of proteins. Our results, showing variability concentrated on antigenic regions on the protein’s surface, is in agreement with previously reported data based on *L. major* and *L. infantum* sequence analysis where regions of GP63 sequence variation were mapped to the surface of the protein and were associated with immunodominant epitopes [37]. However, more studies are needed to better understand the recognition of different GP63 paralogs by the host immune system.

Overall the data presented here highlights novel and relevant aspects related to the expansion of GP63 genes in *L. braziliensis* and related *Viannia* species and raises specific issues regarding the role of GP63 in the parasite pathogenesis during the infection in mammals. It is possible that species belonging to the subgenus *Viannia* may have added a new level of complexity to GP63 function and this may somehow be related to the capacity of some species to cause the more aggressive mucocutaneous form of the disease. The new questions raised here then, when solved, shall provide novel and relevant knowledge regarding the very unique mechanisms of pathogenesis associated with these parasites.

## Conclusions

Our results suggest a greater role for the sequence variation found among the chromosome 10 GP63 genes for the pathogenesis of *L. braziliensis* and closely related species within the mammalian host. The variation in sequence and the expansion in number of these GP63 genes have occurred independently in different *Leishmania* lineages, is associated with intragenic recombination events and has a likely role against the host immune response. They also indicate different functions associated to genes mapped to different chromosomes and, for the chromosome 10 genes at least, variable subcellular localizations likely associated with multiple functions and target substrates for this versatile protease.

## Methods

### Parasites and culture conditions

In this study, we used *Leishmania (Viannia) braziliensis* (MHOM/BR/75/M2904) in its promastigote form. This is a reference strain from the Evandro Chagas Institute, Belém, Brazil. The cells were cultured at 26 °C in Schneider (Sigma) pH 7.2 supplemented with 20% fetal bovine serum (FBS), antibiotics (Streptomycin / Penicillin 0.1%) and 0.1% Hemin.

### PCR, cloning and sequencing

Approximately, 10^8^ *L. braziliensis* promastigotes were used for total genomic DNA extraction using DNAzol (Invitrogen) and standard procedures. PCR reactions for the amplification of the GP63 sequences were performed using Phusion® High-Fidelity DNA Polymerase (New England Biolabs), following the manufacturer’s protocol and with the oligonucleotides used as primers listed in the Additional file 5: Table S5. After amplification, cloning and sequencing of the PCR products, a nomenclature was created for the newly generated sequences in order to identify from which set of primers they were derived, whether from those encoding the KDELMAP or GPI regions, and defining which annotated GP63 gene it most closely resembles. The newly generated sequences derived from the PCR amplifications were deposited on the GenBank and all accession numbers are listed in Additional file 3: Table S3.

### Search for new GP63 paralogs through hidden Markov models

First, the predicted proteomes of the following organisms were downloaded from TritrypDB in August 25, 2014: *L. braziliensis* strain 2903 [taxid: 1295825], *L. braziliensis* strain 2904 [taxid: 420245], *L. infantum* [taxid: 435258]*, L. major* [taxid: 347515]*, L. donovani* [taxid: 981087]*, L. mexicana* [taxid: 929439] and *L. tarentolae* [taxid: 5689]. GP63 genes were then identified within the downloaded proteomes, considering only genes annotated as GP63, encoding proteins longer than 30 amino acids and with no more than one stop codon per sequence. All of the protein sequences derived from genes that met these inclusion criteria were submitted to the analysis of the OrthoMCL program [48], and grouped according to homology using the Markov Cluster algorithm [49]. Protein sequences from each group were aligned using the MAFFT software (default settings) [50] and the multiple alignments used as input for the hmmbuild, version 3.0, a tool from the HMMER package [51] to build Hidden Markov Models (HMMs). The models were then used with the hmmsearch tool to search for new paralogs within the *L. braziliensis* strain 2904 proteome. A cutoff of 0.001 for hit significance (e-value < = 0.001) was applied.

### GP63 phylogenetic analysis and detection of recombination events

A phylogenetic tree was built with GP63 protein sequences from genes encoded within chromosomes 10, 28 and 31 from diverse *Leishmania* species and more distantly related organisms. These include the *Phytomonas* sp. isolate Hart11 [taxid: 134014], *Crithidia fasciculata* [taxid: 5656], *Leptomonas pyrrhocoris* [taxid: 157538], *Trypanossoma cruzi* [taxid: 353153], *Trypanossoma brucei* [taxid: 185431], *Trypanossoma theileri* [taxid: 67003] and *Bodo saltans* [taxid: 75058]. Another tree was made with selected GP63 sequences used in the previous analyses plus the ones obtained by PCR from *L. braziliensis* as well as GP63 sequences from *Leishmania guyanensis* [taxid: 5670]. For all trees, the selected sequences were aligned by MAFFT (default settings) and the alignments automatically edited by Trimal [52] to keep just phylogenetically informative sites. ProtTest [53] was then used to predict the best evolutionary model which was subsequently used as a setting to build phylogenetic trees with PhyML, applying the Maximum Likelihood (ML) method [54], and MrBayes, applying the Bayesian method [55, 56]. The branch support for the ML tree was given by non-parametric bootstrap analysis using 1000 replicates. The Bayesian inferred trees were determined by 5,000,000 chains to check for convergence and a 100% burn-in was discarded. The aligned nucleotide sequences from *L. braziliensis,* obtained from the TriTrypDB database and through PCR, were analyzed for recombination using the RDP4 program [57].

### Modelling of GP63 homologs and searches for non-conserved regions

Eight of the most variable paralogs from different *L. braziliensis* C-terminal groups were chosen for the three-dimensional modelling. The modelling was performed for the amino acid sequences previously obtained from TriTrypDB and applying the SWISS MODEL platform [58]. When the models were completed, their qualities were assessed through Procheck [59]. Specific regions of the protein models were then evaluated using the initial alignment information, highlighting the non-conserved regions which were characterized by amino acid exchanges.

### B-cell epitope prediction

Linear B-cell epitope predictions were performed for the protein sequences used in the 3D modeling step. The predictions were carried out using the following programs: AAP12 [60], BCPred12 [61] and BepiPred [62]. Only epitopes predicted by at least two programs, with lengths equal to or greater than 10 amino acids and with scores greater than 0.8 were considered as positive predictions on AAP12 and BCpred12. Epitopes with scores over 0.5 obtained by BepiPred were also included in the analysis.

In addition to the linear prediction, a conformational prediction of epitopes was also performed to evaluate if the protein structures were also able to generate interaction with the immune system. The conformational epitopes were predicted by the CBTOPE web server [63], where only epitopes with more than 10 amino acids and a score above 4 were considered for this study. After the prediction, an assessment was performed to map the localization of all the epitopes on the modeled proteins.

## Additional files

Additional file 1: **Table S1.** Subsets of GP63 sequences used to build the Hidden Markov Models (HMMs) (DOCX 13 kb)

Additional file 2: **Table S2.** GP63 genes identified by HMM from the *L. braziliensis* M2904 proteome**.** Table showing the number of GP63 genes identified by each HMM after the search for new paralogs within the *L. braziliensis* M2904 genome sequences. (DOCX 11 kb)

Additional file 3: **Table S3.** Set of oligonucleotides, GP63 sequences obtained by PCR and their respective GenBank accession number. (DOCX 15 kb)

Additional file 4: **Table S4.** Table showing the recombination events found for the *L. braziliensis* GP63 genes, followed by the programs that detected it and their *p*-value. Detailed description of the recombination events throughout the *L. braziliensis* GP63 genes. (XLSX 16 kb)

Additional file 5: **Table S5.** Oligonucleotides used for the PCR reactions. A list of the oligonucleotides used for the PCR reactions in this study. (DOCX 13 kb)

## Abbreviations

CL: Cutaneous Leishmaniasis
FBS: Fetal Bovine Serum
GP63: Glycoprotein 63
GPI: GlycosylPhosphatidylInositol
HMMs: Hidden Markov Models
MAC: Membrane Attack Complex
MCL: Mucocutaneous Leishmaniasis
MSP: Major Surface Protease
NPT: Nucleo de Plataformas Tecnológicas
UTR: Untranslated Region
VL: Visceral Leishmaniasis
