# Supplementary material for: In silico characterization of multiple genes encoding the GP63 virulence protein from Leishmania braziliensis: identification of sources of variation and putative roles in immune evasion
Source: BMC Genomics. 2019 Feb 7;20:118. doi: 10.1186/s12864-019-5465-z (PMC6367770; doi:10.1186/s12864-019-5465-z)
Supplement: Supplementary file 1 — Table S1. Subsets of GP63 sequences used to build the Hidden Markov Models (HMMs) (DOCX 13 kb) [file 12864_2019_5465_MOESM1_ESM.docx]

**Table S1. Subsets of Gp63 sequences used to build the Hidden Markov Models (HMMs).** Lbr (*Leishmania braziliensis*), Lmj (*L. major*), Lin (*L. infantum*), Ldn (*L. donovani*), Lmx (*L. mexicana*), Lta (*L. tarentolae*).

| **Subset** | **Number of genes** | ***Leishmania* species (chromosome)** |
| --- | --- | --- |
| 1 | 56 | Lbr (Chr. 10), Lmj (Chr. 10), Lin (Chr. 10), Lmx(Chr. 10), Lta (Chr. 10) |
| 2 | 5 | Lmj (Chr. 28), Lin (Chr. 28), Ldn (Chr. 28), Lmx (Chr. 28) |
| 3 | 9 | Lbr (Chr. 31), Lmj (Chr. 31), Lin (Chr. 31), Ldn (Chr. 31), Lmx (Chr. 30), Lta (Chr. 31) |
| 4 | 3 | Lbr (Chr. 31) |
| 5 | 2 | Lbr (Chr. 10) |
| 6 | 2 | Lta (Chr. 10) |
| 7 | 2 | Lta (Chr. 10) |
| 8 | 2 | Lta (Chr. 10) |
| 9 | 2 | Lta (Chr. 10) |
