# Supplementary material for: In silico characterization of multiple genes encoding the GP63 virulence protein from Leishmania braziliensis: identification of sources of variation and putative roles in immune evasion
Source: BMC Genomics. 2019 Feb 7;20:118. doi: 10.1186/s12864-019-5465-z (PMC6367770; doi:10.1186/s12864-019-5465-z)
Supplement: Supplementary file 2 — Table S2. GP63 genes identified by HMM from the L. braziliensis M2904 proteome. Table showing the number of GP63 genes identified by each HMM after the search for new paralogs within the L. braziliensis M2904 genome sequences. (DOCX 11 kb) [file 12864_2019_5465_MOESM2_ESM.docx]

**Table S2. GP63 genes identified by HMM from the *L. braziliensis* M2904 proteome.** Table showing the number of GP63 genes identified by each HMM after the search for new paralogs within the *L. braziliensis* M2904 genome sequences.

| **HMM** | **Number of GP63 genes identified in *L. braziliensis* strain M2904** |
| --- | --- |
| 1 | 38 |
| 2 | 38 |
| 3 | 38 |
| 4 | 34 |
| 5 | 30 |
| 6 | 31 |
| 7 | 31 |
| 8 | 31 |
| 9 | 31 |
