# Supplementary material for: In silico characterization of multiple genes encoding the GP63 virulence protein from Leishmania braziliensis: identification of sources of variation and putative roles in immune evasion
Source: BMC Genomics. 2019 Feb 7;20:118. doi: 10.1186/s12864-019-5465-z (PMC6367770; doi:10.1186/s12864-019-5465-z)
Supplement: Supplementary file 3 — Table S3. Set of oligonucleotides, GP63 sequences obtained by PCR and their respective GenBank accession number. (DOCX 15 kb) [file 12864_2019_5465_MOESM3_ESM.docx]

**Table S3. Sets of oligonucleotides, GP63 sequences amplified by PCR and their corresponding GenBank accession numbers.**

| **Oligonucleotides (primers) pairs** | **Sequences amplified *** | **Corresponding GenBank Accession number** |
| --- | --- | --- |
| A - 10.0540 (forward); KDELMAP (reverse) | K1610A1; K1610A2; K1650A1 | MH352546, MH352547, MH352548 |
| B - 10.0470 (forward);  GPI (reverse) | G0510B2; G0560B1; G0560B2; G1610B3; G1610B4; G1610B5; G1610B6; G1620B1 | MH352553, MH352552, MH352557, MH352550, MH352551, MH352558, MH352570, MH352549 |
| C - 10.0540 (forward);  GPI (reverse) | G0510C1; G0510C2; G0540C1; G0560C4; G1640C1; G1640C2 | MH352555, MH352573, MH352571, MH352572, MH352554, MH352559 |
| D - 10.0590/0610 (forward); GPI (reverse) | G0590D1; G0590D2 | MH352556, MH352574 |
| F - 10.0406 (forward); KDELMAP (reverse) | K0510F4; K1630F1; K1650F2; K1650F3 | MH352563, MH352561, MH352562, MH352560 |
| G - 10.1516 (forward);  GPI (reverse) | G0510G2; G0520G1; G0560G3 | MH352566, MH352565, MH352564 |
| H - 10.1516 (forward); KDELMAP (reverse) | K1620H2; K1620H3; K1650H4 | MH352567, MH352569, MH352568 |
| I - 10.0470 (forward); KDELMAP (reverse) | K0510I3; K1620I2; K1640I3; K1650I5 | MH376760, MH352567, MH376761, MH376762 |
| K - 31.2260 (forward);  GPI (reverse) | G2260K1 | MH376763 |

* The letter "K" refers to the KDELMAP region, while "G" indicates that the sequence ends at the GPI site. The four-digit number represents the last four numbers of the TriTrypDb GP63 gene that best matches the amplified sequence. The following letter refers to the primer pair used, shown on the left column. Since different sequences had as best matches the same gene, the last number discriminates these sequences. The colours (red and blue) highlight identical sequences.
