# Supplementary material for: In silico characterization of multiple genes encoding the GP63 virulence protein from Leishmania braziliensis: identification of sources of variation and putative roles in immune evasion
Source: BMC Genomics. 2019 Feb 7;20:118. doi: 10.1186/s12864-019-5465-z (PMC6367770; doi:10.1186/s12864-019-5465-z)
Supplement: Supplementary file 5 — Table S5. Oligonucleotides used for the PCR reactions. A list of the oligonucleotides used for the PCR reactions in this study. (DOCX 13 kb) [file 12864_2019_5465_MOESM5_ESM.docx]

**Table S5. Oligonucleotides used for the PCR reactions.**

| **Oligonucleotide** | **Nucleotide sequences** | **Amino acid sequences** |
| --- | --- | --- |
| 0470-F | 5’-ATGTCCCGCGACCGCAGCGT-3’ | MSRDRS |
| 0540-F | 5’-ATGTCCCGCGACCGCAGCAG-3’ | MSRDRS |
| 0590-F | 5’-ATGCCCCTCGACAGCAGCAG-3’ | MPLDSS |
| 1516-F | 5’-ATGTCCCGCGACCGCAGCAGC-3’ | MSRDRSS |
| 0456-F | 5’- ATGYCCCKCGACMGCAGCAG-3’ | MXXDXS |
| GPI-R | 5’-GGCCTGGCACACCTCCACGTA-3’ | YVEVCQA |
| KDELMAP-R | 5’- AGGCGCCATGAGCTCGTCCTT-3’ | KDELMAP |
| 31-F | 5’- ATGTCGCGCGTACCCGTAGCGT-3’ | MSRVPVA |
| 31-GPI-R | 5’- CGCGTAGCACACATCGTCGTAGGGC-3’ | PYDDVCYA |
| 31-KDELMAP-R | 5’-AGGTGCCATCAGCTCGTCCTTCGC-3’ | KDELMAP |
